# Interaction of the mu-opioid receptor with GPR177 (Wntless) inhibits Wnt secretion: potential implications for opioid dependence

**DOI:** 10.1186/1471-2202-11-33

**Published:** 2010-03-09

**Authors:** Jay Jin, Saranya Kittanakom, Victoria Wong, Beverly AS Reyes, Elisabeth J Van Bockstaele, Igor Stagljar, Wade Berrettini, Robert Levenson

**Affiliations:** 1 Department of Pharmacology, Pennsylvania State University College of Medicine, Hershey, PA 17033 USA; 2 Terrence Donnelly Centre for Cellular and Biomolecular Research, Department of Biochemistry and Department of Molecular Genetics, University of Toronto, Toronto ON, Canada M5S 3E1 USA; 3 Department of Neurosurgery, Farber Institute for Neurosciences, Thomas Jefferson University, Philadelphia, PA 19107, USA; 4 Department of Psychiatry, Center for Neurobiology and Behavior, University of Pennsylvania School of Medicine, Philadelphia, PA 19104 USA

## Abstract

**Background:**

Opioid agonist drugs produce analgesia. However, long-term exposure to opioid agonists may lead to opioid dependence. The analgesic and addictive properties of opioid agonist drugs are mediated primarily via the mu-opioid receptor (MOR). Opioid agonists appear to alter neuronal morphology in key brain regions implicated in the development of opioid dependence. However, the precise role of the MOR in the development of these neuronal alterations remains elusive. We hypothesize that identifying and characterizing novel MOR interacting proteins (MORIPs) may help to elucidate the underlying mechanisms involved in the development of opioid dependence.

**Results:**

GPR177, the mammalian ortholog of *Drosophila *Wntless/Evi/Sprinter, was identified as a MORIP in a modified split ubiquitin yeast two-hybrid screen. GPR177 is an evolutionarily conserved protein that plays a critical role in mediating Wnt protein secretion from Wnt producing cells. The MOR/GPR177 interaction was validated in pulldown, coimmunoprecipitation, and colocalization studies using mammalian tissue culture cells. The interaction was also observed in rodent brain, where MOR and GPR177 were coexpressed in close spatial proximity within striatal neurons. At the cellular level, morphine treatment caused a shift in the distribution of GPR177 from cytosol to the cell surface, leading to enhanced MOR/GPR177 complex formation at the cell periphery and the inhibition of Wnt protein secretion.

**Conclusions:**

It is known that chronic morphine treatment decreases dendritic arborization and hippocampal neurogenesis, and Wnt proteins are essential for these processes. We therefore propose that the morphine-mediated MOR/GPR177 interaction may result in decreased Wnt secretion in the CNS, resulting in atrophy of dendritic arbors and decreased neurogenesis. Our results demonstrate a previously unrecognized role for GPR177 in regulating cellular response to opioid drugs.

## Background

Chronic exposure to opioid agonist drugs, such as morphine and heroin, leads to opioid dependence. Opioid agonists cause pronounced changes in neuronal structure and synaptic organization, and these may contribute to the drug craving, compulsive drug use, and analgesic tolerance that characterize the addictive state [1]. Many of these changes reflect inhibitory growth effects of morphine and other opioid agonists on neuron size, neurite outgrowth, dendritic arborization, and neurogenesis that occur in brain regions important for reward processing, learning, and memory [2-5]. However, the specific molecular mechanisms that link opioid agonist activity to the neuronal alterations observed in opioid dependence remain enigmatic.

Most clinically relevant opioid agonists provide analgesia via activation of mu-opioid receptors (MORs), which are members of the G-protein coupled receptor (GPCR) superfamily [6,7]. In many individuals, MOR activation also produces a euphoric effect, which is often considered the primary impetus (or motivational reward) for abusing opioid drugs [8]. MOR knockout mice display significantly reduced sensitivity to both the analgesic and rewarding effects of opioids, thus confirming the importance of the MOR in analgesia and opioid dependence [9,10]. MORs, like most GPCRs, are regulated by multiple mechanisms including receptor desensitization, internalization, degradation, and recycling [11,12]. A number of studies have shown that MOR desensitization and trafficking represent key aspects in the development of opioid tolerance and dependence [8,13-15]. Elucidating the mechanisms that regulate MOR signaling and trafficking is therefore critical for determining the physiological basis of opioid dependence and enhancing opioid receptor pharmacology for the treatment of pain and addiction.

A growing body of evidence indicates that GPCR signaling is modulated by proteins that bind to the GPCR and form multiprotein signaling complexes or signalplexes [16,17]. A number of proteins that interact directly with the mu-opioid receptor have recently been identified and shown to affect MOR biogenesis, trafficking, and signaling [17]. For example, periplakin, protein kinase C interactor1 (PKCI1), and filamin A have all been found to bind to the C-terminal intracellular tail of the MOR. Periplakin inhibits G-protein activation when MOR is bound by an agonist [18], while PCKI1 inhibits agonist-induced phosphorylation of MOR by G-protein coupled receptor kinase 2 [19]. After phosphorylation of MOR, beta-arrestin 2 binds and mediates MOR internalization [20]. Filamin A, on the other hand, serves to anchor MOR to the cytoskeleton by forming a cross-bridge with actin [21].

To gain a better understanding of the potential role of MOR interacting proteins (MORIPs) in mediating opioid dependence, we propose to delineate novel constituents of the MOR signalplex. Using a modified split-ubiquitin yeast two-hybrid approach [22-24], we identified GPR177, a putative orphan GPCR, as a MORIP. Several recent studies have shown that GPR177 is the mammalian ortholog of *Drosophila *Wntless/Evi/Sprinter, an evolutionarily conserved protein that plays an important role in secretion of Wnt proteins from Wnt-producing cells [25-27]. Wnt signaling mechanisms are of particular interest due to their effects on neuronal development [28]. We find that in MOR-expressing HEK293 cells, morphine treatment promotes formation of MOR/GPR177 complexes. This in turn results in sequestration of GPR177 and inhibition of Wnt protein secretion. Defining the constituents of the MOR signalplex thus represents a critical step in understanding the mechanisms of MOR-mediated signaling and the molecular mechanisms underlying opioid dependence.

## Results

### Interaction of the μ-Opioid Receptor (MOR) with GPR177

To identify novel MOR interacting proteins (MORIPs), we performed a modified split-ubiquitin membrane yeast two-hybrid (MYTH) screen [22-24] using the full-length MOR as bait. The MYTH screen uses the split-ubiquitin approach, in which the reconstitution of two ubiquitin halves is mediated by a specific protein-protein interaction [29]. We screened 6 × 10^6 ^colonies and isolated 104 positive clones representing 10 distinct human proteins. Two of them were found to encode the C-terminal domain of GPR177 (residues 451-541). GPR177 is the mammalian ortholog of *Drosophila *Wntless/Evi/Sprinter, a putative multi-pass membrane-spanning protein that is involved in regulating secretion of Wnt proteins from Wnt producing cells [25-27].

**Figure 1:**
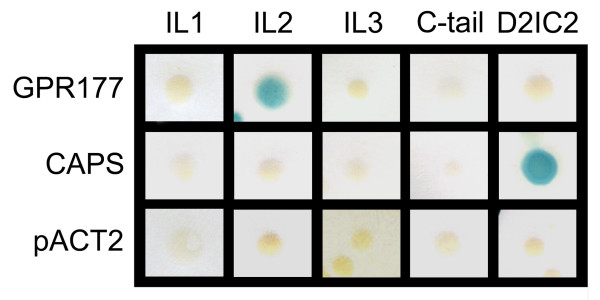
**Directed Y2H screen**. The carboxyl-terminus of GPR177 (residues 451-541) was used as bait in a directed Y2H assay for interaction with each of the intracellular loops (IL) and carboxyl-terminus (C-tail) of the mu-opioid receptor (MOR) and the second intracellular loop of the D2 dopamine receptor (D2IC2). Full-length CAPS (calcium activated protein for secretion), a known interactor of the D2IC2 was used as a positive control, and empty bait vector (pACT2) was used as a negative control. A positive interaction is indicated by the production of a blue colony in the β-gal assay.

Because the split-ubiquitin screen we conducted utilized the entire coding region of the MOR as bait, this approach provided no information regarding the physical location of GPR177 binding sites on the receptor. We therefore used a directed yeast-two hybrid (Y2H) assay to further test for MOR/GPR177 interactions and to map the location of GPR177 binding sites on the MOR. Each of the MOR intracellular loops (IL), including the C-terminal domain (C-tail), were used separately as baits to screen for interaction with the GPR177 clone identified in the initial split-ubiquitin screen. These mapping studies, shown in Figure 1, indicate that GPR177 interacts only with the second intracellular loop (IL2) of the MOR. We also used the Y2H system to examine the specificity of the MOR/GPR177 interaction. In control experiments, the empty prey vector (pACT2) did not cause autologous activation of the reporter gene. GPR177 did not interact with the second intracellular loop of the D2 dopamine receptor (D2IC2), which shares sequence homology with the MORIL2 at five of 22 positions. Calcium-dependent activator protein for secretion (CAPS), a previously identified D2IC2-specific binding protein [30], interacted only with the D2IC2, but not with any of the MOR intracellular domains (Figure 1). These results indicate that GPR177 interacts specifically with the MORIL2 domain in the yeast system.

**Figure 2:**
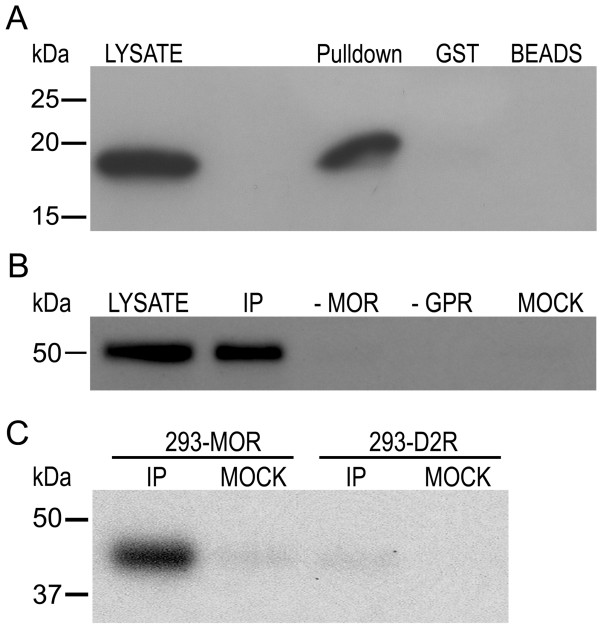
**GPR177 interacts with MOR**. **(A) **GST Pulldown. A MORIL2-GST fusion protein was used to pull down S-tagged GPR177 C-terminal fragment from a bacterial lysate. The 18 kDa S-tagged GPR177 fragment produced in bacteria is shown in the lysate lane, while the 18 kDa S-tagged pull-down product is shown in the pulldown lane. No bands were detectable using beads alone or GST-coated beads. **(B) **Coimmunoprecipitation. The MOR was immunoprecipitated from 293-MOR cells overexpressing FLAG/6× His-tagged GPR177 using an anti-MOR antibody. Immunocomplexes were probed for the presence of GPR177 using chicken anti-GPR177 antibodies. An immunoreactive band of ~50 kDa was detected in the lysate and IP lanes, but not in immunocomplexes from either wild-type HEK293 cells (-MOR lane), 293-MOR cells that were not transfected with GPR177 (-GPR lane), or in IPs in which no anti-MOR antibodies were added (MOCK lane). **(C) **Specificity of MOR/GPR177 interaction. MOR was immunoprecipitated from 293-MOR cells, and the D2 dopamine receptor immunoprecipitated from 293-D2R cells, using anti-FLAG antibodies. Immunocomplexes were probed for the presence of GPR177 using chicken anti-GPR177 antibodies. For all three panels, molecular weight markers (kDa) are shown at the left. Data shown is representative of at least five separate experiments.

To validate the interaction between GPR177 and the MOR, we tested the ability of GPR177 to associate with a MORIL2-GST fusion protein in a pulldown assay. As shown in Figure 2A, a Western blot containing lysate from bacteria expressing an S-tagged GPR177 cDNA fragment (encoding residues 451-541) produced an immunoreactive band of ~18 kDa when probed with anti-S-tag antibodies. This band corresponds to the expected size of the C-terminal GPR177 fragment encoded by the cDNA construct. The same band was detected by pulldown after the bacterial lysate was incubated with the MORIL2-GST fusion protein, but not when the lysate was absorbed onto beads alone or GST-coated beads.

The interaction between full-length MOR and GPR177 was verified in co-immunoprecipitation (CoIP) experiments. To demonstrate interaction, we tested the ability of an anti-MOR antibody to coimmunoprecipitate GPR177 from lysates prepared from HEK293 cells stably expressing FLAG-tagged MORs (293-MOR cells) and transiently transfected with a FLAG/6× His-tagged GPR177 construct. 293-MOR cells were a generous gift from Dr. Mark von Zastrow (UCSF). Preliminary Western blot analysis (Additional file 1, Figure S1A) revealed that the anti-MOR antibody specifically immunoprecipitated (IP) the mu-opioid receptor from 293-MOR cells. Lysates from transfected cells were immunoprecipitated using the anti-MOR antibody, and immunocomplexes probed with chicken anti-GPR177 antibodies. As shown in Figure 2B, an immunoreactive band of ~50 kDA, the predicted size of GPR177, was detected in lysates prepared from transfected cells with the anti-GPR177 antibody. A band migrating with the identical molecular mass was detected in the IP lane, but not in IPs from cells lacking MOR expression (-MOR lane), IPs from 293-MOR cells not transfected with GPR177 (-GPR lane), or mock IPs (mock lane). The specificity of the anti-GPR177 antisera was ascertained by transfecting a FLAG/6× His-tagged GPR177 cDNA into HEK293 cells. A Western blot containing lysate prepared from transfected cells was first probed with anti-FLAG antibodies, then stripped and reprobed with anti-GPR177 antibodies. A band migrating with the identical molecular mass was detected with anti-FLAG and anti-GPR177 antibodies (Additional file 1, Figure S1B), indicating that the GPR177 antibodies react specifically with GPR177.

To examine the specificity of the MOR/GPR177 interaction, we asked whether GPR177 could be coimmunoprecipitated from 293-D2R cells. 293-D2R cells (gift from Dr. Mark von Zastrow) stably express FLAG-tagged D2Rs but not the MOR, and endogenously express GPR177. As shown in Figure 2C, when lysates from 293-MOR cells were immunoprecipitated with anti-FLAG antibodies and immunocomplexes probed with the anti-GPR177 antibody, we detected an immunoreactive band of ~45 kDa. This band corresponds to the size expected for endogenously expressed GPR177 (lacking the FLAG/6 × His tag). In contrast, when lysates prepared from 293-D2R cells were immunoprecipitated with anti-FLAG antibodies, we were unable to detect any immunoreactive bands corresponding to GPR177 when the immunocomplexes were probed using anti-GPR177 antibodies. Taken together, our data strongly suggests that GPR177 interacts specifically with the MOR through the interaction of the carboxy-terminus of GPR177 with the second intracellular loop of the MOR. In the future, direct binding of these two proteins can be established using biophysical-type approaches.

### MOR and GPR177 Interact in PC12 Cells and Rat Brain

**Figure 3:**
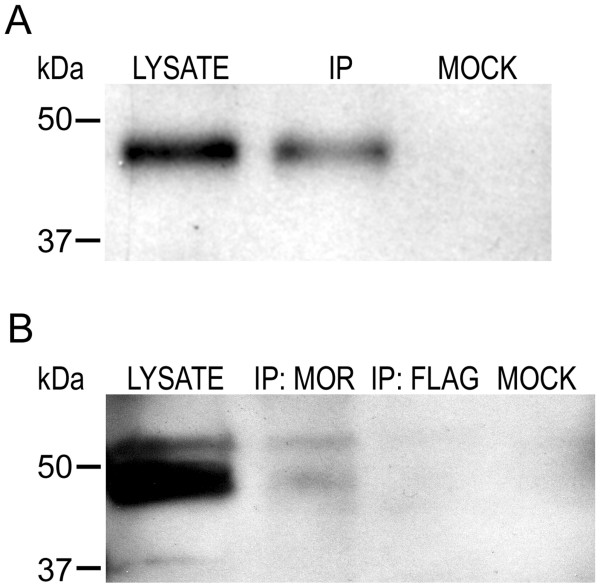
**Interaction of MOR and GPR177 in PC12 cells and rat brain**. **(A) **Lysates prepared from PC12 cells were immunoprecipitated with anti-MOR antibodies. **(B) **Lysates were prepared from total rat brain and immunoprecipitated with anti-MOR antibodies. In **A **and **B**, immunocomplexes were separated by SDS-PAGE, transferred to a PVDF filter, and filters probed with anti-GPR177 antibodies. Molecular weight markers (kDa) are shown at the left. The upper band in rat brain lysates is non-specific. Data shown is representative of at least three separate experiments.

To further characterize the GPR177/MOR interaction, we examined expression of GPR177 in rat pheochromocytoma (PC12) cells and rat brain. Western blotting experiments revealed endogenous expression of GPR177 polypeptides in PC12 cell and rat brain lysates (Figure 3), while PC12 cells have previously been shown to express MORs [31,32]. CoIP experiments were therefore performed to test whether endogenously expressed GPR177 and MORs are associated in PC12 cells and rat brain. To do this, MORs were immunoprecipitated from either PC12 cells or rat brain with anti-MOR antibodies, and immunocomplexes probed for the presence of GPR177. In PC12 cells, anti-GPR177 antibodies reacted with an ~48 kDa band in the lysate and IP lanes, but not in mock immunoprecipitations in which anti-MOR antibodies were omitted (Figure 3A). In rat brain, Western blot analysis revealed the presence of an ~48 kDa band in the lysate and IP lanes, but not in mock immunoprecipitations or immunoprecipitations using non-specific anti-FLAG antibodies (Figure 3B). These results indicate that endogenously expressed GPR177 and MORs interact in PC12 cells as well as rat brain.

### MOR and GPR177 Colocalize in Striatal Neurons

**Figure 4:**
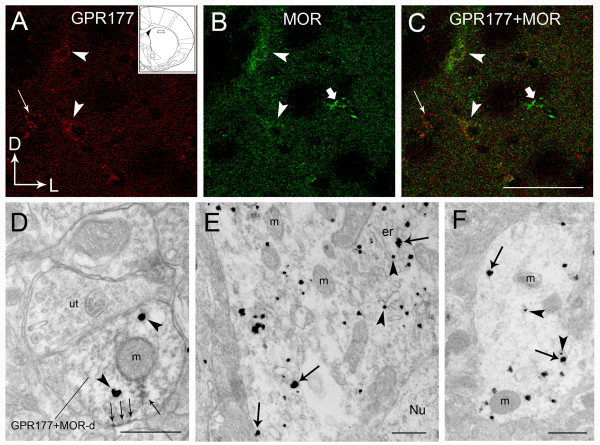
**MOR and GPR177 colocalize within mouse striatum**. Region of the striatum selected for immunohistochemical analysis is shown in the inset of Panel A. **(A-C) **Confocal images of immunolabeling of GPR177 (red) and MOR (green) within striatum. Arrowheads in **A **indicate GPR177 immunolabeling; arrowheads in **B **indicate MOR immunolabeling; arrowheads in **C **(merged image) indicate colocalization of the two proteins. Thin arrows **(A and C) **indicate a cell expressing only GPR177, whereas thick arrows **(B and C) **indicate a cell expressing only MOR. Arrows in lower left corner of panel **A **indicate dorsal (D) and lateral (L) orientations. Scale bar = 0.05 μm. **(D) **Electron photomicrograph showing immunogold-silver (GPR177; arrowheads) and immunoperoxidase (MOR; arrows) labeling within a dendritic process in mouse striatum. An unlabeled terminal (ut) forms a synaptic contact with a dendrite double-labeled for GPR177 and MOR (MOR-d). Scale bar = 50 μm. **(E-F) **Electron photomicrographs showing dual immunogold-silver-labeled GPR177 (arrowheads) and MOR (arrows) within a perikaryon (E) and dendrite (F) within mouse striatum. Scale bar = 0.5 μm. er: Endoplasmic reticulum; Nu: Nucleus; m: Mitochondria.

We next used a combination of immunofluorescence and immunoelectron microscopy to analyze the cellular and subcellular distribution of GPR177 and the MOR in mouse striatum. In double-labeling experiments with anti-GPR177 and anti-MOR antibodies, somatodendritic processes exhibiting coexpression of GPR177 (red) and MOR (green) proteins were localized to common cellular profiles in striatum (Figure 4A-C). These observations suggest that GPR177 colocalizes with the MOR in a subpopulation of striatal neurons.

Double immunoelectron microscopic analysis was used to gain insight into the subcellular distribution of GPR177 and the MOR. For these ultrastructural studies, peroxidase labeling was used to localize MORs and immunogold-silver labeling was used to localize GPR177. As shown in Figure 4D, peroxidase labeling for the MOR was visualized as a diffuse reaction product that was associated with the plasmalemma and cytoplasmic portions of dendritic processes. GPR177 immunoreactivity, visualized as irregularly shaped black deposits indicative of immunogold-silver labeled particles (Figure 4D), exhibited a predominantly cytoplasmic localization.

Expression of GPR177 and the MOR in striatal sections was also analyzed using dual immunogold labeling. As shown in Figure 4E-F, the MOR was identified by large (> 0.05 μm cross-sectional diameter) immunogold-silver particles and GPR177 by smaller (< 0.05 μm cross-sectional diameter) immunogold-silver particles. In double-labeling experiments, GPR177 and MOR immunoreactivity was found in some cases to colocalize in perikarya (Figure 4E) and in dendritic processes (Figure 4F). Within double-labeled structures, some of the immunometal particles representing MOR and GPR177 were situated in close proximity to one another either in the cytoplasm or at the plasma membrane. Taken together, our immunofluorescence data indicate that GPR177 and MORs are coexpressed within the same striatal neuron. The close spatial proximity of the MOR and GPR177 revealed by immunoelectron microscopy provides additional evidence supporting an interaction between these two proteins within neurons.

### Exposure to Opioid Agonists Promotes Formation of MOR/GPR177 Complexes

**Figure 5:**
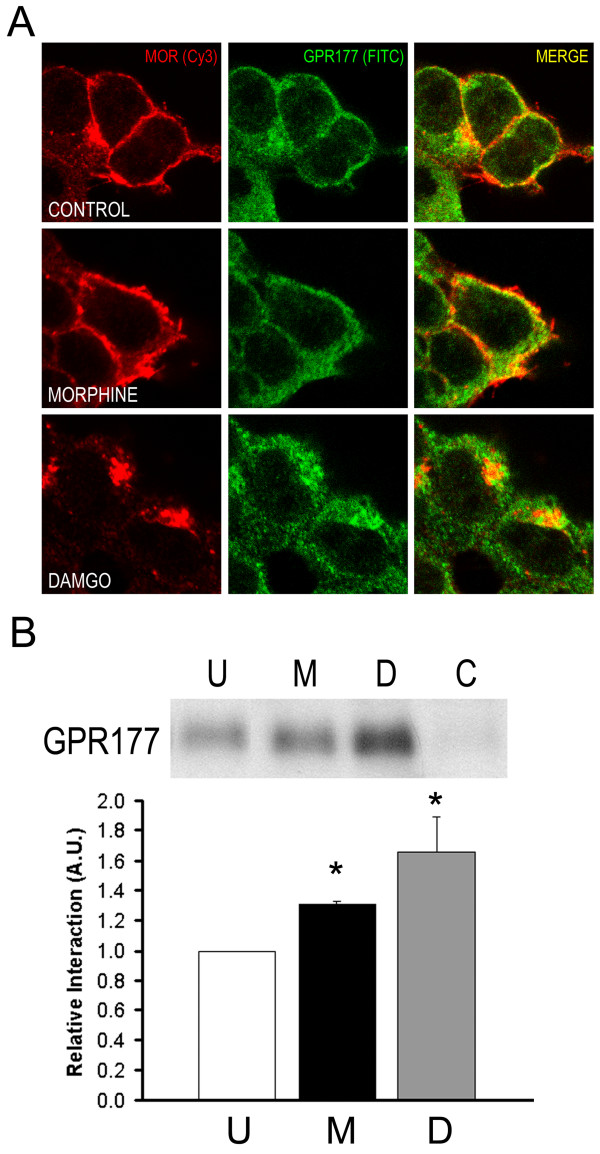
**Effect of opioid agonists on MOR/GPR177 interaction**. **(A) **293-MOR cells were grown either in the absence of drugs (upper panels), or in the presence of 10 μM morphine (middle panels) or 10 μM DAMGO (lower panels) for one hour. Cells were stained with anti-MOR (red) and anti-GPR177 (green) antibodies. Merged images are shown in the right-hand panels. **(B) **293-MOR cells were treated with agonist as in **A**. Cell lysates were immunoprecipitated with anti-MOR antibodies, and immunocomplexes probed with anti-GPR177 antibodies. The bands were analyzed by densitometry and quantitated using ImageJ software. Open bars represent untreated cells, black bars represent morphine-treated cells, and grey bars represent DAMGO-treated cells. Data was analyzed using paired Student's t-test (expressed as mean ± SEM; n = 3, *p < 0.01).

To investigate the effect of opioid agonists on MOR/GPR177 complex formation, we first used confocal laser microscopy to examine the distribution of MOR and GPR177 polypeptides in 293-MOR cells treated with 10 μM morphine for one hour. In untreated and morphine-treated 293-MOR cells, MORs were localized predominantly at the plasma membrane (Figure 5A, left-hand panels). In both untreated and morphine-treated cells, staining for GPR177 was also detected at the periphery, although a considerable amount of GPR177 staining appeared cytosolic (Figure 5A, middle panels). In cells treated with morphine, we observed an increase in MOR/GPR177 colocalization (Figure 5A, merged images), suggesting that morphine treatment may cause an increase in the association between the MOR and GPR177. The distribution of MOR and GPR177 polypeptides in cells treated with 10 μM DAMGO, however, was considerably different. After a one hour exposure period, both the MOR (bottom left panel) and GPR177 (bottom middle panel) appeared to be internalized and staining was predominantly cytosolic. The merged image (bottom right panel) indicates that the bulk of MOR/GPR177 complexes colocalized within cytosolic compartments.

To examine the effect of opioid agonists on the MOR/GPR177 interaction in greater detail, we used coimmunoprecipitation to investigate whether opioid treatment promotes MOR/GPR177 complex formation. In these experiments, 293-MOR cells were exposed to 10 μM morphine or 10 μM DAMGO for one hour. Total cellular lysates were prepared, and the MOR immunoprecipitated using an anti-MOR antibody. Immunocomplexes were then probed for GPR177 with chicken anti-GPR177 antibody. As shown in Figure 5B, MORs were associated with GPR177 under basal conditions, and MOR/GPR177 complex formation appeared to be significantly increased after treatment with morphine or DAMGO. Morphine-induced MOR/GPR177 complex formation was not accompanied by an increase in expression of GPR177 polypeptides (data not shown). Together, these results suggest that both morphine and DAMGO increase MOR/GPR177 complex formation. Further, morphine treatment appears to cause a shift in the distribution of GPR177 from cytosol to the cell surface. This redistribution permits enhanced MOR/GPR177 complex formation at the cell periphery. DAMGO, on the other hand, results in MOR internalization, thus leading to an increased association between the MOR and GPR177 within the cytosol.

### Effect of MOR/GPR177 Interaction on Wnt secretion

**Figure 6:**
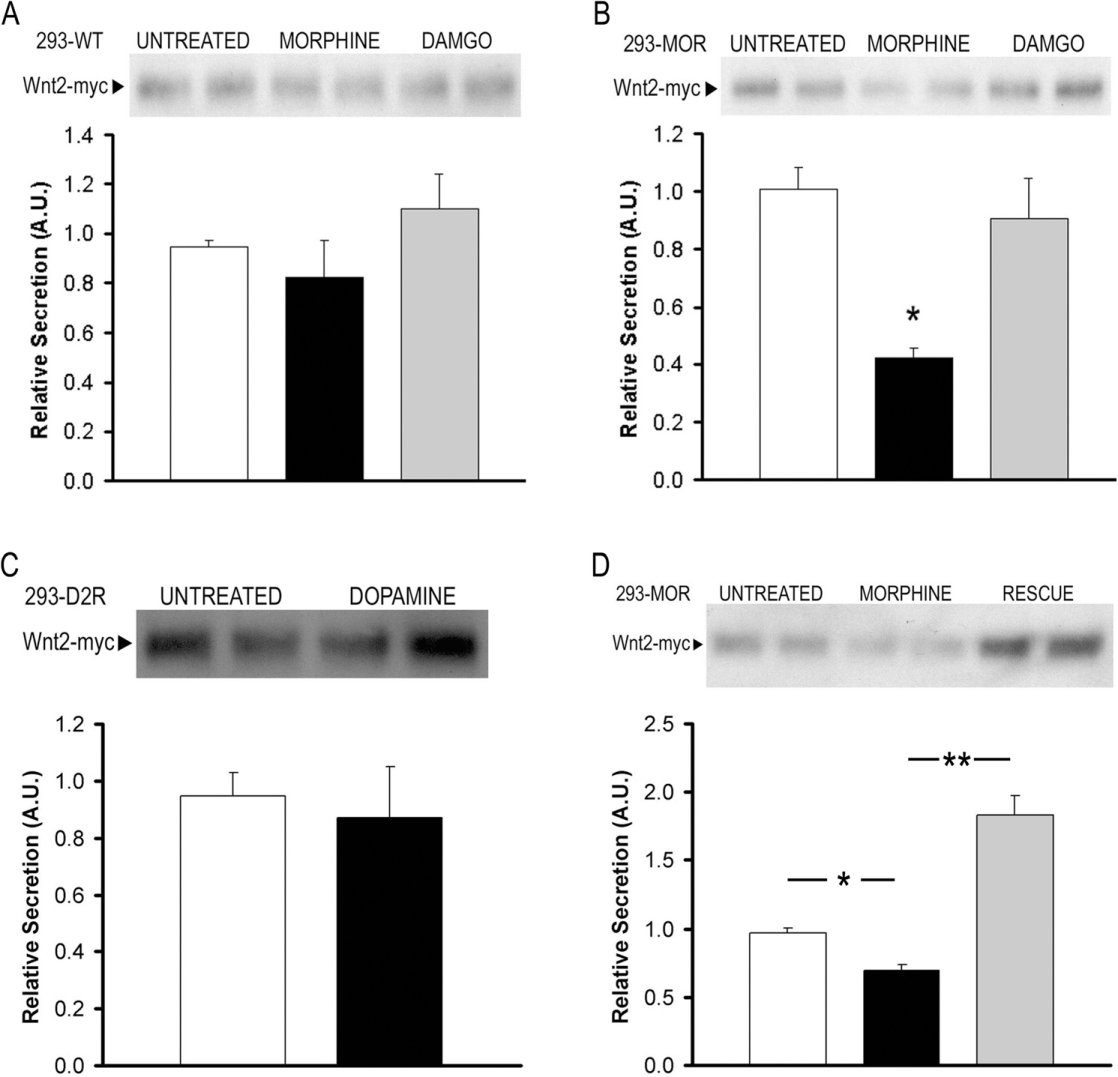
**Morphine inhibits Wnt secretion**. Wild-type HEK293 **(A) **and 293-MOR cells **(B) **were transfected with myc-tagged Wnt2 cDNA. 24 hours after transfection, cells were treated with either 10 μM morphine or 10 μM DAMGO for an additional 24 hours. In **A **and **B**, open bars represent non-drug-treated controls, black bars represent morphine-treated cells, and grey bars represent cells treated with DAMGO. **(C) **293-D2R cells were transfected and processed as in **A **and **B**, but were cultured in the presence of 10 μM dopamine instead of morphine. Media was collected, proteins separated by SDS-PAGE, then transferred to a PVDF filter. Filters were probed with anti-myc antibodies. Open bars represent non-drug-treated controls, black bars represent drug-treated cells. **(D) **Overexpression of GPR177 rescues Wnt secretion. 293-MOR cells were transfected with myc-tagged Wnt2 and GPR177 cDNAs. Cells were processed and proteins visualized as described above. Open bars represent no drug treatment, black bars represent morphine-treated cells, and grey bars represent cells overexpressing GPR177. Bands were analyzed by densitometry and quantitated using ImageJ software. Data was analyzed using a two-sided Student's t-test (expressed as mean ± SEM; n= 4-5; *p < 0.05; **p < 0.01).

Recent studies have shown that GPR177 plays an essential role in mediating Wnt protein secretion from Wnt-producing cells [25-27]. We therefore wished to determine whether the interaction between GPR177 and MOR might contribute to the regulation of Wnt secretion. To test this idea, we examined Wnt secretion utilizing HEK293 cells, which do not normally express Wnt proteins, but do secrete Wnt polypeptides after transient transfection with Wnt cDNA [25,26]. As shown in Figure 6 (panels A-D), wild-type HEK293, 293-MOR, and 293-D2R cells are all capable of secreting Wnt2 polypeptides after transient transfection with myc-tagged Wnt2 cDNA. Treatment of wild-type HEK293 cells with morphine or DAMGO produced no significant effect on Wnt2 secretion compared to untreated cells (Figure 6A). However, treatment of 293-MOR cells with morphine produced a significant decrease in Wnt2 secretion compared to untreated controls (Figure 6B). The inhibition of Wnt secretion appears to be specific to the activation of MORs by morphine, since DAMGO did not affect Wnt2 secretion from 293-MOR cells (Figure 6B). Further, dopamine treatment did not significantly affect Wnt2 secretion from D2 dopamine receptor-expressing 293-D2R cells (Figure 6C).

We next asked whether the morphine-mediated inhibition of Wnt2 secretion could be reversed by overexpression of GPR177. To do this, we cotransfected 293-MOR cells with myc-tagged Wnt2 and GPR177 cDNAs, and monitored Wnt2 secretion in cells grown in the presence or absence of morphine. As shown in Figure 6D, morphine treatment caused a significant inhibition of Wnt2 secretion compared to untreated controls. However, overexpression of GPR177 in morphine-treated 293-MOR cells produced a dramatic increase in Wnt2 secretion. Taken together, the data from these experiments strongly suggests that morphine treatment specifically blocks Wnt secretion from MOR expressing cells, and that this inhibition can be overcome by overexpression of GPR177.

**Figure 7:**
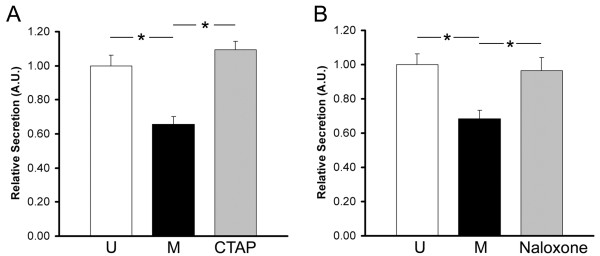
**Effect of MOR antagonists on Wnt secretion**. 293-MOR cells were transfected with myc-tagged Wnt2 cDNA. 24 hours after transfection, cells were treated with either **(A) **10 μM CTAP (D-Phe-Cys-Tyr-D-Trp-Arg-Thr-Pen-Thr-NH2) or **(B) **10 μM naloxone for one hour followed by an additional 23 hours of treatment with 1 μM morphine. Media was collected, proteins separated by SDS-PAGE, then transferred to a PVDF filter. Filters were probed with anti-myc antibodies and immunoreactive bands scanned by laser densitometry and quantitated using ImageJ software. In **A **and **B**, open bars represent untreated 293-MOR cells, black bars represent 293-MOR cells treated with morphine, and grey bars represent 293-MOR cells treated with either CTAP plus morphine or naloxone plus morphine. Data was analyzed using a two-sided Student's t-test (expressed as mean ± SEM; n= 5-6; *p < 0.05).

To confirm that the inhibition of Wnt2 secretion by morphine is regulated through the mu-opioid receptor, we tested the ability of the potent MOR antagonists CTAP [33] and naloxone [34], to block the morphine-mediated effect. In these experiments, 293-MOR cells were pretreated with either CTAP or naloxone for one hour prior to the addition of morphine and the initiation of the secretion assay. As shown in Figure 7, pretreatment with either CTAP or naloxone reversed the morphine-mediated inhibition of Wnt2 secretion. These results provide compelling evidence that the morphine-mediated inhibition of Wnt2 secretion is regulated via the mu-opioid receptor.

## Discussion

In this study, we demonstrate that mu-opioid receptors (MORs) can functionally interact with GPR177, an evolutionarily conserved protein that mediates Wnt protein secretion in a variety of species including flies, worms, frogs, rodents, and humans [35-37]. Through its interaction with the MOR, GPR177 appears to play an important physiological role in neuronal response to opioid agonists. This interpretation is based on the following conclusions. First, the MOR/GPR177 interaction occurs within neurons. Second, the association between these two proteins is enhanced by morphine treatment. Third, an increase in the interaction between the MOR and GPR177 results in the inhibition of Wnt secretion. These findings shed new light on the role of MORIPs in the neurobiology of opioid agonist actions, and offer a mechanism whereby chronic opioid agonist exposure may produce deleterious effects on neuronal structure and function via inhibition of Wnt secretion.

The interaction between the MOR and GPR177 was initially uncovered in a MYTH screen and then validated in GST-pulldown and coimmunoprecipitation assays. To our knowledge, this represents the first successful MYTH screen using a full-length human GPCR as bait. The potential physiological relevance of the MOR/GPR177 interaction is borne out by the data in Figures 3 and 4. The coimmunoprecipitation results presented in Figure 3 indicate that MORs and GPR177 endogenously expressed within cultured cells and brain tissue are capable of interacting with each other. The double-label light and immunoelectron microscopic analysis shown in Figure 4 further establish that MOR and GPR177 are coexpressed within a subset of striatal neurons. The close spatial proximity of MOR and GPR177 immunoreactivity within perikarya and dendrites provides compelling evidence for an interaction between these two proteins within striatal neurons. Since GPR177 and MOR are widely expressed in rodent and human brain (unpublished observations) it is likely that the MOR/GPR177 interaction occurs in many brain regions.

**Figure 8:**
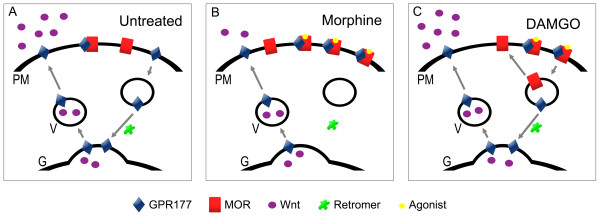
**Proposed model for effect of MOR/GPR177interaction on Wnt secretion**. **(A) **Under basal conditions, MOR and GPR177 are associated at the plasma membrane (PM) and in recycling vesicles (V). However, GPR177 is located primarily in vesicles in association with Wnts, whereas MOR is predominantly plasma membrane-associated. Recycling of GPR177 from PM to Golgi (G) is mediated via retromer, and is required for Wnt secretion. **(B) **In the presence of morphine, MORs are inefficiently internalized which leads to an increase in the association between MOR and GPR177 near the plasma membrane. This results in reduced retromer-mediated GPR177 recycling and functional sequestration of GPR177 activity. Reduced recycling of GPR177 leads to inhibition of Wnt secretion. **(C) **In the presence of DAMGO, MORs are efficiently internalized which allows for internalization of GPR177 proteins associated with MORs. Once internalized, GPR177 is recycled to the Golgi apparatus via retromer, thereby allowing GPR177 to associate with Wnt proteins and functionally support Wnt protein secretion.

Our analysis of the effect of opioid agonists on MOR/GPR177 complex formation, together with the Wnt secretion experiments, indicate that the MOR/GPR177 interaction is likely to be of importance for understanding the changes that occur in brain after chronic opioid use. We now propose a model (Figure 8) in which the MOR/GPR177 interaction regulates the secretion of Wnt proteins that are required for neuronal development and morphogenesis. This model is based on the following lines of evidence. GPR177 is a protein that mediates Wnt secretion in a variety of vertebrate and invertebrate species [35]. GPR177 has also been shown to regulate Wnt secretion in cultured *Drosophila *and mammalian cells [25,26]. Using immunohistochemistry and coimmunoprecipitation, we found that the level of MOR/GPR177 complexes was significantly increased after treatment of 293-MOR cells with morphine. This observation is consistent with data reported in other systems. In mice, for example, opioid agonist activation of MOR causes a significant increase in the association of MOR with spinophilin, a dendritic spine-enriched scaffold protein [38].

Our data indicate that MOR and GPR177 are binding partners, and their association with one another occurs in cytosolic compartments as well as at the cell surface. GPR177 has previously been shown to interact with Wnt proteins in the Golgi, and the two proteins traffic together to the plasma membrane where Wnt proteins are then secreted [25]. GPR177 is internalized via clathrin-mediated endocytosis and recycled back to the Golgi via the retromer complex [39,40]. In the Wnt secretion experiments we performed, treatment of wild-type HEK293 cells with either morphine or DAMGO produced no significant effect on Wnt secretion compared to untreated control cells (Figure 6A). In untreated 293-MOR cells (Figure 6B), the MOR/GPR177 interaction that takes place under basal conditions does not appear to interfere either with the ability of GPR177 to mediate Wnt secretion nor with normal GPR177 trafficking.

When cells are treated with morphine, however, the situation is quite different. Our results indicate that morphine application appears to cause a redistribution of GPR177 from cytosol to the cell surface and enhanced MOR/GPR177 complex formation at the cell periphery. In morphine-treated 293-MOR cells, this leads to an inhibition of Wnt secretion (Figure 6B). Since it is well established that morphine application leads to delayed MOR internalization [41-45], we hypothesize that the morphine-enhanced interaction between MOR and GPR177 causes entrapment of GPR177 at the cell surface, effectively sequestering GPR177 and inhibiting its proper function in mediating Wnt secretion. We propose that when MOR is bound to GPR177, GPR177 is unable to shuttle Wnt proteins between the Golgi and the cell surface (Figure 8B). However, as has been shown previously, DAMGO application leads to fast internalization of MORs [41,43,45]. Thus, DAMGO treatment may allow for rapid internalization of GPR177. In our model (Figure 8C), we propose that following DAMGO treatment, MOR/GPR177 complexes internalize from clathrin-coated pits into early endosomes. After internalization, MOR/GPR177 complexes dissociate, and GPR177 is recycled to the Golgi via association with the retromer complex. In the Golgi, GPR177 is able to associate with Wnt proteins that are then targeted to the plasma membrane for secretion [25-27]. Because GPR177 cycling is likely to be maintained in the presence of DAMGO, there is no significant effect of DAMGO on Wnt secretion.

Our data demonstrate a previously unrecognized role for GPR177, a novel MORIP, in regulating cellular response to opioid agonist drugs. Morphine treatment induces cell-surface associated MOR/GPR177 complex formation which in turn inhibits Wnt protein secretion. Wnt proteins have been known for some time to play an essential role in morphological patterning during development [46]. New roles for these proteins have also been discovered in stem cell maintenance [47,48], cancer biology [49], as well as neuronal development [28] and synaptic remodeling [50,51]. In brain, Wnt3 has been shown to regulate neurogenesis in hippocampus [52], while Wnt7b mediates dendritic arborization [53]. These processes are known to be severely compromised after chronic morphine exposure [4,54]. Our discovery of an interaction between the MOR and GPR177 may thus represent an important conceptual breakthrough in understanding the mechanism whereby opioid agonist drugs produce these profound morphological changes in brain. The loss of dendritic arborization and/or neurogenesis may be key to understanding the myriad problems associated with opioid use including addiction, cognitive dysfunction, depression, and impaired visual and motor skills. Clearly, the ability to determine the influence of GPR177 on the acute and chronic actions of opioids will require the development of appropriate mouse models in which the effect of disrupting the GPR177 gene can be analyzed in behavioral paradigms of opioid agonist action. The molecular actions of GPR177 suggest that it could serve as a novel drug target that could be utilized to promote analgesia while preventing the deleterious neuronal alterations that accompany chronic opioid agonist use.

## Conclusions

In this study, we demonstrate an interaction between the MOR and GPR177, an evolutionarily conserved protein that regulates secretion of Wnt proteins in Wnt-producing cells. The interaction between these two binding partners is enhanced by treatment of cells with opioid agonist drugs, and results in the inhibition of Wnt protein secretion. Based on these findings, we propose a model in which opioid agonist inhibition of Wnt protein secretion is responsible for the loss of dendritic arborization and neurogenesis that occurs after chronic opioid exposure. These findings suggest a previously unrecognized role for GPR177 in regulating cellular response to opioid agonist drugs, and could create new avenues of investigation regarding the pharmacotherapy of drug dependence.

## Methods

### Split-Ubiquitin Membrane and Conventional Yeast Two-Hybrid Screens

A modified split-ubiquitin membrane yeast two-hybrid (MYTH) screen was performed as previously described [22-24]. Briefly, full-length human MOR cDNA in the bait vector pCCW-STE (Dualsystems Biotech AG, Switzerland) and human fetal brain cDNA library in the prey vector pPR3-N (Dualsystems) were sequentially transformed into *S. cerevisiae *reporter strain THY.AP4. Transformation of yeast with the human brain library produced 6 × 10^6 ^transformants/μg DNA on synthetic dropout (SD) agar plates (-Trp/-Leu/-His/-Ade; Clontech, Palo Alto, CA) containing 3-amino-1,2,4-triazole (3AT). From this screen we identified 10 novel MORIPs, two of which contained cDNAs encoding the C-terminal tail (residues 451-541) of GPR177.

To identify sites of interaction between MOR and GPR177, each MOR intracellular loop (IL) was tested for interaction with the carboxyl-terminus of GPR177 using the conventional yeast two-hybrid (Y2H) method. MOR IL domains (IL1, residues 97-103; IL2, residues 166-187; IL3, residues 260-282; C-tail, residues 342-400) were separately ligated into the yeast GAL4 DNA-binding domain vector pAS2-1 (Clontech), while the carboxyl-terminus of GPR177 (GPR177-CT: residues 451-541) was ligated into the yeast GAL4 activation domain vector pACT2 (Clontech). Bait and prey plasmids were simultaneously cotransformed into *S. cerevisiae *strain MAV103 as previously described [55]. Transformants were identified by growth on SD/-Trp/-Leu agar plates. Interactions were assayed for β-galactosidase activity via the nitrocellulose filter lift method [55].

### Cell Culture and Drug Treatment

Human Embryonic Kidney (HEK) 293 cells and rat pheochromocytoma (PC12) cells were maintained in Dulbecco's Modified Eagle's Medium (DMEM) supplemented with 10% fetal bovine serum. FLAG-tagged MOR expressing HEK293 (293-MOR) cells and FLAG-tagged D2 dopamine receptor (D2R) expressing HEK293 (293-D2R) cells were provided by Dr. Mark von Zastrow (University of California, San Francisco) and maintained in DMEM supplemented with 10% fetal bovine serum and 400 μg/ml geneticin (Invitrogen, Carlsbad, CA). Transfections were carried out using Effectene transfection reagent (Qiagen, Valencia, CA) according to manufacturer's instructions. Morphine sulfate (10 μM; Baxter Healthcare, Deerfield, IL), [D-Ala^2^, N-MePhe^4^, Gly-ol]-enkephalin (DAMGO, 10 μM; Sigma, St. Louis, MO), and dopamine (10 μM; Sigma) were diluted directly into cell culture medium.

### Antibody Production and Immunocytochemistry

Anti-GPR177 antibodies were raised in chickens against a peptide antigen corresponding to the C-terminal 18 amino acids (HVDGPTEIYKLTRKEAQE) of human GPR177 (Gene-Tel Laboratories, Madison, WI). Antibodies of the IgY subtype were harvested from egg yolks and affinity purified prior to use. For immunocytochemistry, 293-MOR cells were grown on poly-L-lysine (Sigma) coated coverslips for 24 hours, treated with drug for one hour, then fixed with 4% paraformaldehyde for 15 minutes. Cells were washed 3 times with phosphate-buffered saline (PBS) then incubated with blocking solution (5% goat serum, 0.1% Triton X-100, 1× PBS) for 1 hour at room temperature. Double labeling was performed by incubation with rabbit anti-MOR (AB5511; 1:500 dilution; Millipore, Bedford, MA) and chicken anti-GPR177 (1:500 dilution) antibodies. Double staining was visualized with Cy3-conjugated goat anti-rabbit (1:500 dilution, Jackson ImmunoResearch, West Grove, PA) and fluorescein isothiocyanate (FITC)-conjugated goat anti-chicken antibodies (1:500 dilution, Jackson ImmunoResearch). Fluorescent images were captured on a Leica TCS SP2 AOBS confocal microscope.

### Glutathione S-transferase Pull-Down and Coimmunoprecipitation

A MORIL2-GST (glutathione S-transferase) fusion protein was constructed by fusing amino acids 166-187 of the human MOR to GST in the expression vector pGEX-4T-1 (Amersham Biosciences, Piscataway, NJ). The MORIL2-GST-fusion protein was induced in *Escherichia coli *strain BL21 (DE3) using the ZYP-5052 auto-induction media described previously [56,57]. The GPR177 C-terminal fragment (residues 451-541) was subcloned into the pET30C expression vector containing an S-tag. Constructs were induced using auto-induction media as described above. MORIL2-GST fusion protein was used to pull down the GPR177 C-terminal fragment from bacterial lysates as previously described [55]. Eluted proteins were separated by SDS-PAGE and transferred to a polyvinylidene fluoride (PVDF) filter. The filter was probed with a horseradish peroxidase (HRP) conjugated S-tag antibody (1:5000 dilution, Novagen, Madison, WI), and immunoreactivity detected by enhanced chemiluminescence with an ECL Plus kit (GE Healthcare, Piscataway, NJ)

For coimmunoprecipitation experiments, a full-length GPR177 cDNA containing an N-terminal 6× histidine (His) tag was ligated into the pCMV-Tag2 mammalian expression vector, and the construct transfected into 293-MOR cells. Immunoprecipitations were performed from total cell lysates as previously described [58]. MORs were immunoprecipitated from lysates using an anti-MOR antibody (AB1580, Millipore). Western analysis of immunoprecipitated complexes was performed by using polyclonal anti-GPR177 antibody (1:5000 dilution). Staining was visualized with a 1:15,000 dilution of HRP-conjugated donkey anti-chicken secondary antibodies (Jackson ImmunoResearch).

### Wnt2 secretion assay

HEK293, 293-MOR, or 293-D2R cells were separately transfected with a myc-tagged human Wnt2 cDNA in the pCMV-Tag3 mammalian expression vector (Stratagene, La Jolla, CA). Cells were grown in DMEM plus 10% FBS for 24 hrs, then grown in the presence or absence of 10 μM morphine, 10 μM DAMGO, or 10 μM dopamine in DMEM lacking FBS, for an additional 24 hrs prior to initiating secretion assays. To assess secretion, medium was collected and concentrated (approximately 22-fold) using an Amicon Ultra-15 centrifugal filter (Millipore). Aliquots of medium (45 μl) were fractionated on SDS-containing 10% polyacrylamide gels. Proteins were transferred to a PVDF membrane, and the filter probed with a monoclonal anti-myc antibody (1:2500 dilution; Millipore). Proteins were visualized using HRP-conjugated rabbit-anti-mouse secondary antibodies (1:15,000 dilution; Jackson ImmunoResearch). Immunoreactivity was detected by ECL using an ECL Plus kit, and scanned blots quantitated using the ImageJ software package (NIH). Data were subjected to a two-tailed Student's t-test.

### Immunofluorescence Microscopy

Mice used for immunohistochemistry were housed and treated according to institutional guidelines. Adult male mice were perfused with 50 ml of 4% formaldehyde in 0.1 M PB; pH 7.4, and brains were removed and post-fixed in 4% formaldehyde overnight at 4°C. Striatal sections (40 μm) were rinsed extensively in 0.1 M PB and 0.1 M tris-buffered saline (TBS; pH 7.6), incubated in 0.5% bovine serum albumin (BSA) and 0.25% Triton X-100 in 0.1 M TBS for 30 min, then rinsed extensively in 0.1 M TBS. Sections were incubated with a cocktail containing chicken anti-GPR177 (1:1,000 dilution) and rabbit anti-MOR (1:2,000 dilution; Immunostar Inc., Hudson, WI) antibodies. Sections were washed in 0.1 M TBS, then incubated in a cocktail containing Alexafluor 488 donkey anti-rabbit (1:80 dilution; Jackson ImmunoResearch) and tetramethyl rhodamine isothiocyanate (TRITC) donkey anti-chicken (1:200 dilution; Jackson ImmunoResearch) secondary antibodies. Fluorescent images were obtained with a confocal microscope (Zeiss LSM 510 Meta, Carl Zeiss Inc., Thornwood, NY), and digital images were captured and imported with the LSM 5 image browser (Carl Zeiss Inc.).

### Immunoelectron Microscopy

Mice were perfused with (1) 10 ml heparinized saline, (2) 50 ml of 4% formaldehyde in 0.1 M PB, pH 7.4. Immediately after perfusion, brains were removed, sectioned into coronal slices and postfixed overnight at 4°C in the same fixative. Alternate 40 μm thick sections through the rostrocaudal extent of the striatum were incubated with a cocktail of chicken anti-GPR177 (1:1,000 dilution) and rabbit anti-MOR (1:2,000 dilution) antibodies. Immunoperoxidase labeling was used to identify MOR immunoreactivity while immunogold-silver labeling was used to identify GPR177. Primary antibodies were complexed with a mixture of biotinylated donkey anti-rabbit (1:400 dilution; Jackson ImmunoResearch) and ultra small (< 1 nm) gold-coupled goat anti-chicken (1:100 dilution; Electron Microscopy Sciences, Fort Washington, PA) secondary antibodies. MOR was detected by incubation with avidin-biotin complex (Vector Laboratories, Burlingame, CA) and visualized by DAB (3,3'-diaminobenzidine; Sigma). To visualize immunogold-labeled GPR177, silver enhancement of the gold particles was performed using a silver enhancement kit (Amersham Bioscience, Piscataway, NJ). Sections were flat embedded in Epon 812 (Electron Microscopy Sciences). Thin sections (50-100 nm) were collected on copper mesh grids and examined with an electron microscope (Morgagni Fei Company, Hillsboro, OR). Digital images were captured using an AMT advantage HR/HR-B CCD camera system (Advance Microscopy Techniques Corp., Danvers, MA).

For dual immunogold labeling, 40 μm sections through the rostrocaudal extent were incubated in a cocktail containing chicken anti-GPR177 (1:1,000 dilution) and rabbit anti-MOR (1:2,000 dilution) primary antibodies. Sections were incubated with ultra small gold-coupled goat anti-rabbit (1:100 dilution; Amersham Bioscience) secondary antibody, followed by the first silver enhancement (300 μl R-Gent SE-EM enhancement mixture; Amersham Bioscience). Sections were then incubated with ultra small gold-coupled goat anti-chicken (1:100 dilution; Amersham Bioscience) secondary antibody, followed by the second silver enhancement (300 μl R-Gent SE-EM enhancement mixture; Amersham Bioscience). Immunogold-silver particles measuring less than 0.05 μm in cross-sectional diameter were scored as small, while immunogold-silver particles measuring greater than 0.051 μm were scored as large. Tissues sections were incubated in 2% osmium tetroxide (Electron Microscopy Sciences) and flat embedded in Epon 812. Digital image capturing was as described above.

## Authors' contributions

JJ generated the GPR177 antibody, carried out all protein interaction studies, immunocytochemistry, Wnt secretion studies, and drafted the manuscript. SK, VW, and IS carried out the split-ubiquitin yeast two-hybrid screen. BASR and EJVB carried out the in vivo immunohistochemistry and electron microscopy. WB participated in the design of the study, formulated hypotheses, and supplied critical reagents for protein interaction studies. RL conceived of the study, and participated in its design and coordination and helped to draft the manuscript. All authors read and approved the final manuscript.

## Supplementary Material

Additional file 1 **Figure S1 - Specificity of anti-MOR and anti-GPR177 antibodies**. **(A) **Specificity of anti-MOR antibodies. 293-MOR cells, stably expressing FLAG-tagged MORs, were transiently transfected with a FLAG/6x His-tagged GPR177 construct. Lysates were immunoprecipitated with anti-MOR antibodies (Millipore). Immunocomplexes were separated by SDS-PAGE, transferred to a PVDF filter, and filters probed with M2 anti-FLAG monoclonal antibodies. Molecular weight markers (kDa) are shown at the left. The transfected GPR177 was detected in the lysate lane, while both GPR177 and the MOR were detected in the IP lane. MORs were also detected in IPs from untransfected 293-MOR cells (-GPR177 lane). No immunoreactive bands were detected in IPs from wild-type HEK293 cells (lacking MORs) transiently transfected with GPR177 (-MOR lane) or in IPs of transiently transfected 293-MOR cells in which anti-MOR antibodies were omitted (Mock lane). **(B) **Specificity of anti-GPR177 antibodies. Lysates were prepared from 293-MOR cells (293-MOR lysate lanes) and 293-MOR cells transiently transfected with a FLAG/6x His-tagged GPR177 construct (+FLAG/His GPR177 lanes). The immunoblot was initially probed with M2 anti-FLAG antibodies (left panel). The blot was then stripped and reprobed with anti-GPR177 antibodies (right panel). Molecular weight markers (kDa) are shown at the left. A band migrating with the identical molecular mass was detected with M2 anti-FLAG and anti-GPR177 antibodies, indicating that GPR177 antibodies react specifically with GPR177. Click here for file
