# Supplementary figures and images for: Interaction of the mu-opioid receptor with GPR177 (Wntless) inhibits Wnt secretion: potential implications for opioid dependence
Source: BMC Neurosci. 2010 Mar 9;11:33. doi: 10.1186/1471-2202-11-33 (PMC2841195; doi:10.1186/1471-2202-11-33)

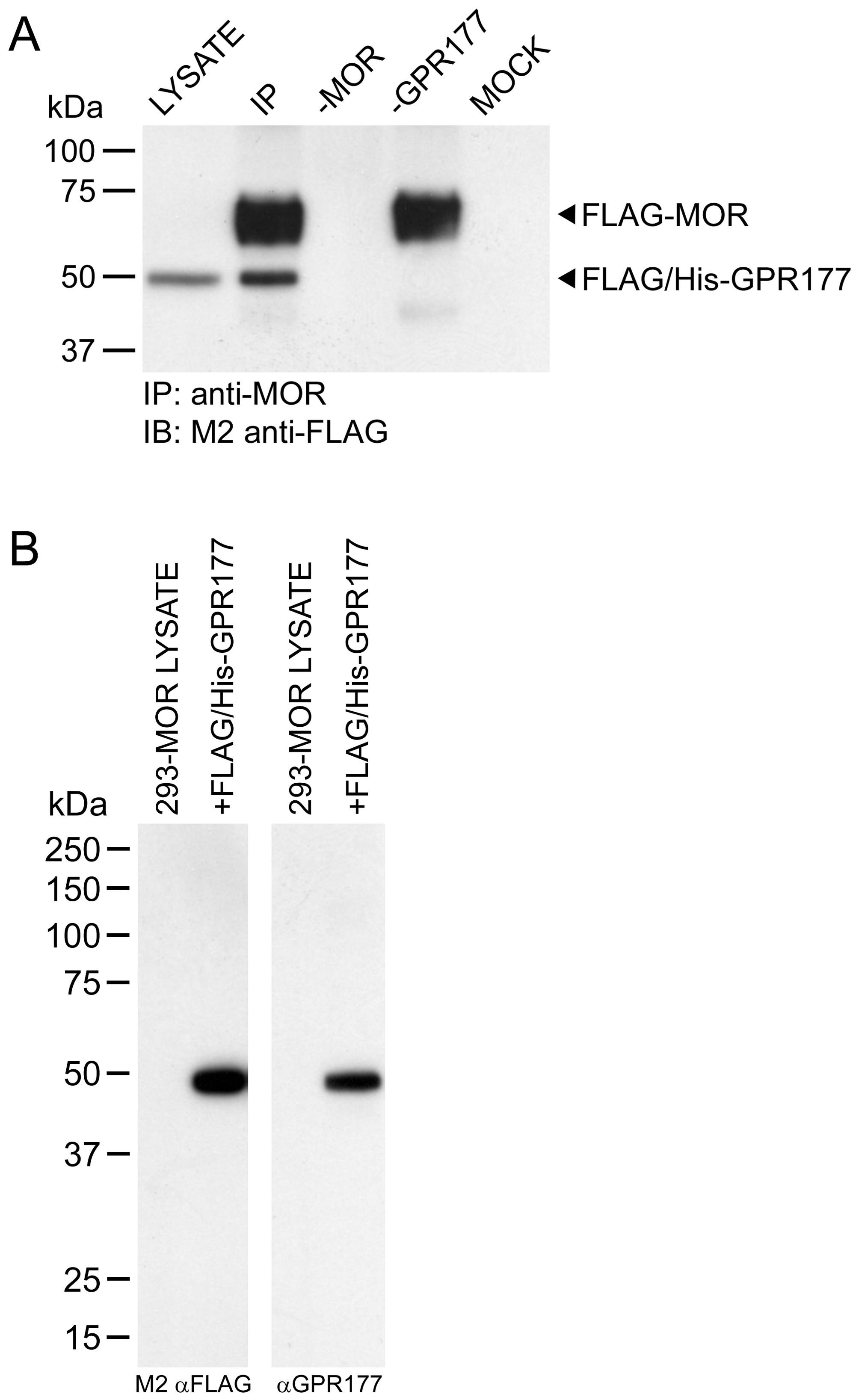

Supplement: Additional file 1 — Figure S1 - Specificity of anti-MOR and anti-GPR177 antibodies. (A) Specificity of anti-MOR antibodies. 293-MOR cells, stably expressing FLAG-tagged MORs, were transiently transfected with a FLAG/6x His-tagged GPR177 construct. Lysates were immunoprecipitated with anti-MOR antibodies (Millipore). Immunocomplexes were separated by SDS-PAGE, transferred to a PVDF filter, and filters probed with M2 anti-FLAG monoclonal antibodies. Molecular weight markers (kDa) are shown at the left. The transfected GPR177 was detected in the lysate lane, while both GPR177 and the MOR were detected in the IP lane. MORs were also detected in IPs from untransfected 293-MOR cells (-GPR177 lane). No immunoreactive bands were detected in IPs from wild-type HEK293 cells (lacking MORs) transiently transfected with GPR177 (-MOR lane) or in IPs of transiently transfected 293-MOR cells in which anti-MOR antibodies were omitted (Mock lane). (B) Specificity of anti-GPR177 antibodies. Lysates were prepared from 293-MOR cells (293-MOR lysate lanes) and 293-MOR cells transiently transfected with a FLAG/6x His-tagged GPR177 construct (+FLAG/His GPR177 lanes). The immunoblot was initially probed with M2 anti-FLAG antibodies (left panel). The blot was then stripped and reprobed with anti-GPR177 antibodies (right panel). Molecular weight markers (kDa) are shown at the left. A band migrating with the identical molecular mass was detected with M2 anti-FLAG and anti-GPR177 antibodies, indicating that GPR177 antibodies react specifically with GPR177. [file 1471-2202-11-33-S1.TIFF]
